# Uteroglobin-related protein 1 and severity of inhalation injury

**DOI:** 10.1186/2197-425X-3-S1-A320

**Published:** 2015-10-01

**Authors:** G Friedman, SF Henrich, TH Rech, F Dal Pizzol

**Affiliations:** Universidade Federal do Rio Grande do Sul, PPG Ciências Pneumológicas, Porto Alegre, Brazil; Universidade Federal do Rio Grande do Sul, Hospital de Clínicas de Porto Alegre, Porto Alegre, Brazil; Universidade do Extremo Sul Catarinense, PPG Ciências da Saúde, Criciúma, Brazil

## Introduction

Burns are a major global public health problem. It is estimated that over 300 million burn victims die each year worldwide. Most accidents are domestic or on workplace and involve mainly women and children. With the advances made in the care of burned patients, the mortality has decreased in recent years and the inhalation injury has become the leading cause of death in these patients. Mortality associated to smoke inhalation alone may be low (0-11%), but the inhalation combined with skin burns can reach 90%. Uteroglobin-related protein 1 (UGRP1) is a secretory protein expressed in the airways, and speculated to have anti-inflammatory activity.

## Objectives

To evaluate if UGRP1 plasma levels are related to the severity of inhalation injury.

## Methods

Observational study, mechanically ventilated patients admitted to the Intensive Care Unit of the Hospital de Clinicas de Porto Alegre, victims of a great fire in January 2013 in the city of Santa Maria, state of Rio Grande do Sul, Brazil.

## Results

We included 16 patients (age: 23 ± 5 yrs), with inhalation injury: grades 1 (n = 3), 2 (n = 4), and 3 (n = 9). UGRP1 levels were significantly related to the severity of inhalation (Grade 1 0.389 ± 0.053 mcg vs. grade 2 0.474 ± .0423 mcg vs grade 3 0.580 ± 0.094 mcg; p = 0.007).

## Conclusions

Uteroglobin-related protein 1 levels are significantly increased in patients with inhalation injury and associated to the injury severity, particularly in those with grade 3 inhalation injury.

## Grant Acknowledgment

Universidade do Extremo Sul Catarinense

